# Congenital nephrotic syndrome and recurrence of proteinuria after renal transplantation

**DOI:** 10.1007/s00467-014-2781-z

**Published:** 2014-03-29

**Authors:** Christer Holmberg, Hannu Jalanko

**Affiliations:** Children’s Hospital, University of Helsinki and Helsinki University Central Hospital, PO Box 281, Helsinki, 00290 Finland

**Keywords:** Congenital nephrotic syndrome, Renephrosis, Anti-nephrin antibodies, Rituximab

## Abstract

Renal transplantation (RTx) is the only curative treatment for most cases of congenital and infantile nephrotic syndrome (NS) caused by genetic defects in glomerular podocyte proteins. The outcome of RTx in these children is usually excellent, with no recurrence of nephrotic syndrome. A subgroup of patients with the Finnish type of congenital nephrosis (CNF), shows, however, a clear risk for post-RTx proteinuria. Most of these patients have a homozygous truncating mutation (Fin-major mutation) in the nephrin gene (*NPHS1*), leading to total absence of the major podocyte protein, nephrin. After RTx, these patients develop anti-nephrin antibodies resulting in nephrotic range proteinuria. Plasma exchange combined with cyclophosphamide and anti-CD20 antibodies has proved to be successful therapy for these episodes. NS recurrence has also occurred in a few patients with mutations in the podocin gene (*NPHS2*). No anti-podocin antibodies have been detectable, and the pathophysiology of the recurrence remains open. While most of these episodes have resolved, the optimal therapy remains to be determined.

## Introduction

Congenital nephrotic syndrome (CNS), defined as severe proteinuria during the first 3 months of life, leads to hypoproteinemia, oliguria, edema, and other consequences of severe protein loss such as hyperlipidemia, hypothyreosis, and risk for thrombotic complications and infections [1]. Most cases of CNS are autosomal recessive diseases caused by genetic defects in different components of the glomerular filtration barrier (primary CNS), especially by mutations in nephrin (NPHS1, nephrotic syndrome type 1) and podocin (NPHS2) genes. Secondary forms of CNS are rare and often caused by treatable infections (congenital syphilis, toxoplasmosis, malaria, cytomegalovirus, rubella).

For most primary forms of CNS, the only curative treatment is kidney transplantation (RTx). The outcome of RTx in the vast majority of patients with primary CNS is excellent with no signs of recurrence of the original disease. In a small minority, however, post-RTx proteinuria develops and forms a diagnostic and therapeutic challenge. It must be emphasized that recurrence of proteinuria after RTx is quite common in non-genetic forms of steroid-resistant nephrotic syndrome (SRNS) [2], which account for the majority (80 %) of SRNS cases in childhood. This review focuses exclusively on the rare cases of recurrence in patients with genetic causes of CNS.

## Genetic causes of CNS

**Fig. 1:**
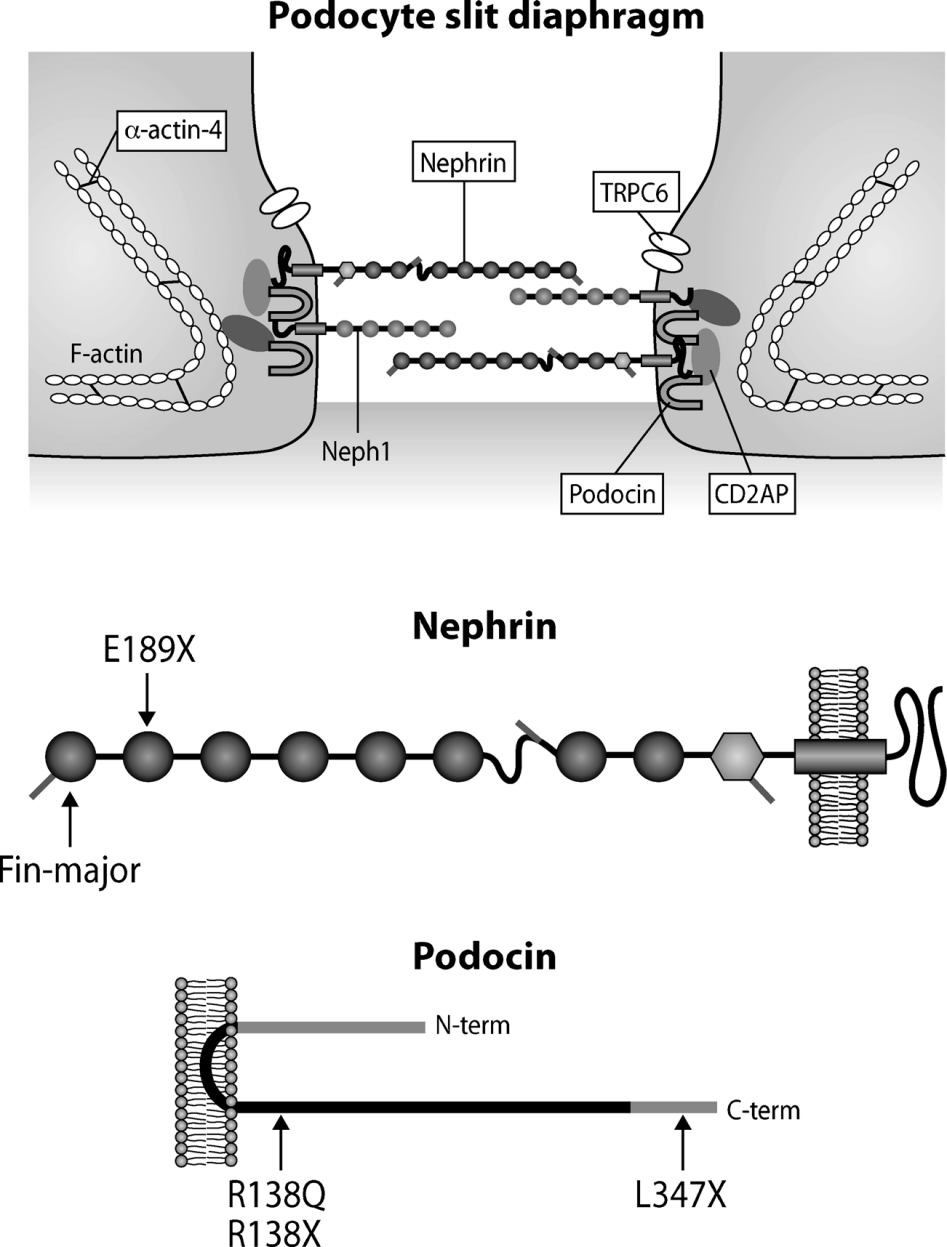
The model of the slit diaphragm (SD) showing some of the major components (*top*). Nephrin is a transmembrane protein with extracellular Ig-domains (*circles*) and one fibronectin type domain (*hexagon*) (*middle*). Fin-major (nt121(del2)) mutation leads to a truncated protein of only 90 residues (out of 1,241 amino acids). E189X mutation also results in severely truncated protein (189 residues). Podocin is an intracellular protein belonging to the stomatin family (*bottom*). Both R138Q and R138X are common mutations leading to a severely truncated podocin protein. Similarly, L347X, which is caused by a single nucleotide deletion (948delT) in the last podocin exon, results in a truncated protein

In 1956, Niilo Hallman et al. reported eight infants with nephrotic syndrome (NS) from birth, resistant to all treatment and leading to death within the first year of life [3]. This entity was called CNS of the Finnish type (CNF) and it is still the prototype for CNS. In 1998, Marjo Kestilä and co-workers [4] isolated the gene responsible for CNF, named it *NPHS1*, and the same group localized its gene product, nephrin, to the slit diaphragm (SD) connecting the podocyte foot processes in the glomerular capillary wall [5]. Nephrin is a transmembrane adhesion protein of the immunoglobulin family containing 1,241 amino acids (Fig. 1). The extracellular part of nephrin has eight immunoglobulin-like motifs and one type III fibronectin domain [4]. Nephrin molecules from neighboring foot processes of separate podocyte cells are thought to interact in the center of the SD through homophilic interactions, thus forming a zipper-like structure. The nephrin molecules also interact at the cell surface with shorter Neph molecules. The intracellular domain has nine tyrosine residues, some of which become phosphorylated during ligand binding. Nephrin takes part in cell signaling and is important in connecting the SD to actin cytoskeleton of the podocyte foot process [6]. Mutations in *NPHS1* usually lead to severe proteinuria from birth, but some patients have their NS onset later in childhood. More than 200 mutations in *NPHS1* have emerged (www.biobase-international.com), most of which lead to a severe clinical form of CNS. The Finnish patients have two founder mutations: Fin-major (nt121(del2)) leading to a truncated 90 residue protein, and Fin-minor (R1109X) leading to a truncated 1,109-residue protein. Approximately two-thirds (63 %) of the Finnish patients have Fin-major/Fin-major genotype, 18 % are Fin-major/Fin-minor compound heterozygotes, and 9 % are Fin-minor/Fin-minor homozygotes [7].

CNF patients must be treated symptomatically with albumin infusions, a protein-rich diet, thyroxin and anticoagulation, and aggressive treatment of septic infections [8]. As long as the patients do well and grow and develop normally, one can just monitor them. However, severely proteinuric patients, especially those with reduced growth and frequent infections, should undergo early nephrectomy and RTx, which can be performed with retroperitoneal placement of the graft when their weight is over 10 kg [1].

In 2000, Boute et al. described another gene, *NPHS2*, encoding for an important podocyte protein, podocin [9]. Mutations in *NPHS2* lead to typical CNS but also to NS manifesting later in life. Podocin is an intracellular linker protein that interacts with nephrin and serves a scaffolding function for the SD. More than 60 pathogenic mutations described can lead to steroid-resistant nephrotic syndrome (SRNS) presenting from birth to adulthood [10]. The R138Q mutation is associated with early onset NS. The histological presentation is usually one of focal segmental glomerulosclerosis (FSGS).

*NPHS1* and *NPHS2* mutations account for about 75 % of the primary CNS cases [11]. They both cause isolated CNS without major extrarenal manifestations. Other important etiologies (Table 1) are phospholipase C epsilon-1 (*PLCE1*, [12]) and Wilms tumor gene 1 (*WT1*, [13]), mutations that typically cause diffuse mesangial sclerosis (DMS) and progressive impairment of renal function. Laminin β2 (*LAMB2*) mutations also cause microcoria and the so-called Pierson’s syndrome [14]. Decaprenyl-diphosphate synthetase subunit 2 (*PDSS2)* and co-enzyme Q2 4-hydroxybenzoate polyprenyltransferase *(COQ2)* mutation cause muscular symptoms of mitochondriopathies [15, 16]. Genetic defects in *ARHGDIA* that encodes Rho guanosine diphosphate dissociation inhibitor-α have recently been shown to cause CNS and neurological handicap [17, 18].

**Table 1:** Some important podocyte genes, mutations of which can lead to congenital nephrotic syndrome (CNS) (11–18)

Gene	Protein	Locus	Function	Phenotype
*NPHS1*	Nephrin	19q13.1	Structural basis of SD, signaling	CNS
*NPHS2*	Podocin	1q25-31	Links nephrin to lipid rafts	CNS, SRNS
			Signaling	FSGS
*TRPC6*	Cation channel	11q21-22	Calcium influx signaling	SRNS, FSGS
*PLCE1*	Phospholipase	10q23	Signaling	SRNS, DMS, FSGS
	Cε1			
*WT1*	Wilms tumor 1	11p13	Differentiation	FSGS, DMS
				Denys-Drash-S
*LAMB2*	Laminin β2	3p21	podocyte to GBM	DMS, Pierson S
*PDSS2*	CoenzymQ synthetase	6q21	CoQ10 production	FSGS, Leigh S
*COQ2*	PHB-propenyl transferase	4q21,23	CoQ10 production	Collapsing glomerulopathy
*ARHGDIA*	RhoGDIα	17q25,3	GPD-diss. inhibitor	FSGS

*SD* slit diaphragm, *S* Syndrome, *SRNS* steroid-resistant nephrotic syndrome, *FSGS* focal segmental glomerulosclerosis, *DMS* diffuse mesangial sclerosis

## Renal transplantation in infants with CNS

Reports from most registries and larger centers show that graft and patient survival after RTx in infants is at least as good as in older children [19]. A recent Canadian study showed that the greatest risk for graft failure was in young adults—not in infants [20]. One Scandinavian study showed results as good in infants as in older patients [21]. Cultural and socioeconomic differences do, however, exist, and results are hard to compare [22, 23]. Today it is clear that early RTx is indicated in CNS patients, as most long-term acquired problems develop during the nephrotic or uremic stage. Perioperative problems in infants are comparable to those in older children and adults. An adult graft, however, can be placed extraperitoneally only after the child weighs about 10 kg. Before that, an intraperitoneal placement of the graft can be considered. Postoperatively, excessive fluids are needed to adequately perfuse the kidney graft [24]. Long-term graft function in infants is similar to that in older children. A recent finding has also shown that growth is good, in fact catch up growth in infants is better [25], puberty is normal, and final height is acceptable in patients transplanted as infants [26]. Neurocognitive function in children without co-morbidities or complications before RTx is satisfactory and family coping is excellent in developed societies with social support [27].

## Proteinuria after RTx

After RTx, mild proteinuria is not rare. The most common causes are chronic allograft injury, de novo glomerulopathy and drug toxicity. In this context, a special problem is non-genetic FSGS; this is a major cause of SRNS and in children accounts for 11 % of end-stage renal disease [28]. Heavy proteinuria recurs in 20-40 % of the patients, often within days after RTx [29]. A circulating plasma factor has been suggested as being responsible, and recent research has suggested that circulating soluble urokinase receptor (suPAR), increased TNF-α activity, or additional factors are involved [30, 31].

## Recurrent proteinuria in NPHS1 patients

In 1992, Laine et al. reported on 28 CNF patients, of whom six (24 %) developed severe proteinuria and NS 1-33 months after RTx [32]. Histology showed endothelial swelling and mesangial cell proliferation. All patients were treated with methylprednisolone (MP) and five with additional cyclophosphamide (CP). Only two patients went into remission, and four grafts were lost. One patient showed proteinuria again in the second graft 14 months after re-transplantation. Three additional CNF patients reported to have proteinuria after transplantation had responded, two to steroids and one to steroids and cyclophosphamide [33–35]. This indicates that a risk for proteinuria in CNF seems to exist after early RTx, with some patients responding to therapy.

In 2000, Patrakka et al. described 45 CNF (NPHS1) patients receiving 51 kidneys [36]. In this Finnish cohort, 15 episodes of recurrent proteinuria occurred in 13 grafts (25 %). All nine patients with recurrence were homozygous for the Fin-major mutation, which leads to an early stop-codon and total absence of nephrin in the native kidney. Rescue therapy with CP was successful in seven episodes, but six kidneys were lost. Antibodies reacting against the glomerulus were found in eight of the nine patients, and high serum anti-nephrin antibody levels were detected by an ELISA method in four. Thus, it seems that circulating anti-nephrin antibodies play a pathogenic role in NS recurrence. This resembles the situation in Alport’s syndrome, in which an immune response against the previously unseen collagen epitopes in kidney grafts is responsible for the de-novo anti-glomerular basement membrane disease [37].

In 2007, Kuusniemi et al. reported 65 Finnish CNF patients who had received 77 kidneys [38]. The recurrence rate in Fin-major/Fin-major patients was then 34 %, with anti-nephrin antibodies found in 8/11 patients (73 %). No anti-nephrin antibodies were detectable before RTx in the 22 patients studied. In patients with a re-transplantation, recurrence occurred in 1–22 days. One patient, after graft removal, showed a clear increase in anti-nephrin antibodies, suggesting that the antibodies might not always be evident in the circulation but can still be present locally. Plasma exchange (PE) was tried in addition to MP and CP in seven patients with nine episodes of NS after transplantation. Only one graft was lost (11 %) in contrast to five (45 %) in the 11 patients treated only with steroids and CP. PE was performed on the five subsequent days and then three times a week depending on response thereafter. CP was given for 12 weeks and then switched to azathioprine or mycophenolate as part of triple immunosuppression. Glomerular filtration rate (GFR) remained as good as in patients without NS recurrence. One patient failing to react to PE also received an anti-CD20 monoclonal antibody (Rituximab) and high-dose immunoglobulin without any response. It thus seems that the recurrence rate is about 30 % in Fin-major/Fin-major patients, that 70 % have measurable anti-nephrin antibodies, and that most patients react to MP and CP combined with PE, and their GFR remains good.

In 2006, Srivastava et al. described a patient with NS 7 days after an RTx from his aunt [39]. He had 3248G > T and 3250delG mutations in exon 24 and a 3478C > T mutation in exon 27 and showed no nephrin expression in the kidney. No anti-nephrin antibodies were present, but as serum permeability activity was high it suggested a circulating factor might play a role in re-nephrosis. In 2012, a Stanford group reported a child with a homozygous mutation in *NPHS1* with transversion G > T at nucleotide position 565 that causes an amino acid change in the nephrin protein: glutamic acid for Amber (stop codon). The child underwent RTx at 2.5 years and developed NS with anti-nephrin antibodies 6 months after transplantation [40]. On biopsy, slight rejection appeared, and CD20-staining was positive. The child was treated with increased steroids and CP, and as no immediate reaction was evident, Rituximab was added. The patient went slowly into remission, and the authors speculate that Rituximab might have contributed to that favorable result.

Table 2 shows our last six NPHS1 patients with recurrent NS. They all received high-dose MP (15–20 mg/kg) and most also CP and PE. Rituximab (eliminating CD20-positive B lymphocytes) was added in five children and two patients also received bortezomib (eliminating antibody-producing plasma cells). All except one went into remission, now lasting for 3.5 to 7.7 years. The only patient not responding developed NS during a H1N1 infection. In patients 2, 3, and 6 (Table 2) Rituximab (375 mg/m^2^ × 2–4 weekly doses) had an immediate effect: inducing permanent remission. The pathogenic role of antibodies is supported by the fact that three children each had two episodes of NS which occurred soon (5–31 days) after re-transplantation, suggesting preformed antibodies.

**Table 2:** Treatment and outcome of the last six Finnish CNS patients with recurrence of proteinuria and nephrotic syndrome after RTx

Patient	Mutation	dgn	RTx, age	Re-nephrosis (no)	Time after RTx (mo)	Therapy	Outcome (+ time since last remission in bold)
1 UTI, polyoma, rejections	Fin-major homozygote	at birth	1y 7mo	1)	51	MP, Cyclo, PE	**Remission** **7y 7mo**
2	Fin-major homozygote	at birth	2y 4mo	1) 2) 3) 4) 5) 6)	4, 5 12 23 26 31 40	MP, Cyclo, PE MP, Cyclo, PE MP, Cyclo, PE MP, Cyclo, PE, ACE MP, Cyclo, PE, ACE MP,** Rituxi x4, **ACE	Remission “ “ “ “ **Remission** **6y**
3	Fin-major homozygote	at birth	1y 1mo	1) 2)	40 42	MP, Cyclo, PE ACE MP, **Rituxi x4** ACE	Remission after 1mo **Remission** **5y 6mo**
4	Fin-major homozygote	at birth	2y 10mo	1) 2)	4 5 20	MP, PE, **Rituxi x4** Cyclo MP, PE, *Bortetsomibi x4* **Rituxi x1**	Remission after 12mo still γ-glob. infusions **Remission 3y 7mo**
5 H1N1	Fin-major homozygote	at birth	1y 4mo	1) 2)	32 60	MP, Cyclo, PE +**Rituxi** **x2** +ACE +*Bortetsomibi x4* +**Rituxi x1** MP, **Rituxi x4** ACE	U-prot: 1.5 g/l Transplant nephropathy, HD
6	Fin-major homozygote	at birth	1y 8mo	1)	13 14	MP, Cyclo, PE **Rituxi** **x2**	Remission after 11mo **Remission 4y 6mo**

*RTx* renal transplantation, *y* year, *mo* month, *MP* methylprednisolone, *Cyclo* cyclophosphamide, *PE* plasma exchange, *ACE* angiotensin-converting enzyme inhibition, *Rituxi* Rituximab, *CNS* congenital nephrotic syndrome, *UTI* urinary tract infection

All Finnish patients with recurrence had severe proteinuria and were Fin-major homozygotes, which is a rare mutation outside Finland. The mutation stops gene reading very early, and thus no tolerance to nephrin develops in these patients during maturation of their immunological system. Nephrin located in the kidney graft is therefore a completely new antigen for Fin-major/Fin-major patients and raises an immunological response. Although in non-Finnish patients a wide variety of missense mutations, insertions and deletions appear, development of a natural tolerance to the nephrin molecule seems to be the rule.

In the management of these patients, our current practice is, as soon as proteinuria is detected, to give three MP-pulses and to start daily PE sessions. Analysis of anti-nephrin antibodies is not routinely performed as the test may give false-negative results. In cases with heavy proteinuria and no previous CP therapies, commencement of oral CP medication (2.5 mg/kg/day) is routine. If no response occurs in 3 weeks, 2–4 doses of Rituximab (375 mg/m^2^) are given, and CP is switched to mycophenolate. In cases with a previous CP exposure, Rituximab is the first option. Our experience with bortezomib is thus far very limited, and we have not tried anti-complement factor five antibodies (eculizumab). The fact that no complement deposits are present in the kidney graft with recurrence speaks against the use of eculizumab.

## Recurrent proteinuria in NPHS2 patients

An *NPHS2* mutation is responsible for FSGS in 18–40 % of affected children; in such children, most studies do not report recurrence after renal transplantation. Post-transplant recurrence of proteinuria in patients affected by homozygous or compound heterozygous NPHS2 mutations is encountered rarely (1–2 %), compared to the 30 % recurrence rate seen in nonhereditary FSGS [29]. However, descriptions exist of five such patients (Table 3).

**Table 3:** Treatment and outcome of CNS patients with NPHS2 and recurrence of proteinuria and nephrotic syndrome after RTx (29, 41–45)

Patient no	Nucleotide change	Exon	Coding sequence	Age at RTx	Proteinuria after RTx	Proteinuria	Treatment	Outcome
1	413G > A 413G > A	3	R138Q R138Q	9 y	10 d	2–3 g/l	PE Cyclo	Good
2	413G > A 413G > A	3	R138Q R138Q	4.5 y	300 d	2–3 g/l	PE Cyclo	Good
3	412C > T 412C > T	3	R138X R138X	3.1 y	4 y	TP/Cr 5.5 g/g	PE	Stable
4	948delT 948delT	8	L347X L347X	4.5 y	7 d	2.4 g/l	MP -pulses	Good
5	412C > T 412C > T	3	R138X R138X		2 y			
6	413G > A IVS4-1,G > T	3 5	R138Q Split	7 y	10 y	10.7 g/m^2^	Switch from sirolimus to CsA	Good

*RTx* renal transplantation, *y* year, *d* day, *PE* plasma exchange, *Cyclo* cyclophosphamide, *MP* methylprednisolone, *CsA* cyclosporine A, *TP* total protein, *Cr* creatinine, *CNS* congenital nephrotic syndrome

Bertelli et al. [29] reported two children homozygous for the R138Q mutation who presented with recurrence of proteinuria 10 and 300 days after RTx. Proteinuria was heavy and occurred when the patients had normal renal function. They were treated with plasma exchange (6–15 cycles) and cyclophosphamide (2 mg/kg for 60 days and four iv pulses). Early and late outcome in both patients was good [29].

Billing et al. reported a patient with a single-nucleotide deletion in exon 8 of *NPHS2* (948delT), for which the patient was homozygous [41]. At the age of 4.5 years, this patient received a renal graft from her mother. On day 7 after RTx, she developed progressive proteinuria (urine protein/creatinine ratio 2.4 g/g), which responded in one week to prednisone pulse therapy and increased CsA dosage. The patient has maintained stable graft function and no recurrence of proteinuria has been observed.

Becker-Cohen et al. described a 9-year-old girl with SRNS presenting at the age of 2 months [42]. She was homozygous for the R138X mutation, which causes truncation of the podocin protein and is the most common cause of familial SRNS among Arab children. Four years after RTx from a deceased donor, significant proteinuria appeared and gradually increased into the nephrotic range. In biopsy, no signs of acute rejection emerged, and a search for anti-podocin antibodies could exclude their presence. Plasma exchange was started three times weekly for 9 months then twice weekly for 2 weeks. No immediate response was documented, immunosuppression was kept unchanged, with no antiproteinuric medication given. However, subsequently proteinuria decreased significantly.

Weber et al. described one patient from a cohort of 32 patients with homozygous or compound heterozygous mutations in *NPHS2* [43]. The patient was homozygous for R138X mutation and developed biopsy-proven FSGS 2 years after a third RTx, this one from her mother. No anti-podocin antibodies appeared in indirect immunofluorescence.

Höcker et al. reported a pediatric RTx patient with a heterozygous R138Q mutation and a heterozygous splice-site mutation (IVS 4-1,G-T) before exon 5, together leading to NPHS2-associated FSGS [44]. She developed biopsy-proven recurrence of FSGS 10 years post-RTx in association with conversion from CsA-based to sirolimus-based immunosuppression. A re-switch of the immunosuppressive regimen back to CsA led to a noticeable decrease in proteinuria and to stabilization of graft function.

SRNS caused by *NPHS2* mutations is regarded as an autosomal recessive disorder requiring mutations in both alleles. In some patients, however, only a solitary heterozygous mutation has been detectable and a few of these patients have demonstrated post-RTx recurrence of proteinuria [29]. It has been postulated that patients with a single mutation have a different underlying pathogenesis of their disease, which could involve defects in other proteins composing the glomerular barrier or a combination of decreased podocin expression together with immunological injury.

In contrast to the situation in NPHS1, the pathomechanism of the recurrent proteinuria in NPHS2 patients remains unknown and probably is multifactorial. As pointed out earlier, no anti-podocin antibodies have been detectable in any of the patients studied. The most common mutations in NPHS2 patients with recurrence have been R138X, which leads to a truncated podocin protein, and R138Q, resulting in a change of a highly conserved arginine to glutamine. Theoretically, immunologic response in a NPHS2 patient with two truncating mutations is reasonable, as the kidney graft presents to the recipient “new” podocin epitopes. On the other hand, immunization seems less likely in a patient with only an amino acid change in the native podocin. Moreover, the fact that podocin is an intracellular podocyte protein makes it a less attractive target for immunological attack as compared to nephrin, which forms the backbone of the extracellular SD.

Post-RTx recurrence of proteinuria in non-genetic FSGS is believed to be caused by a circulating “proteinuric factor”, which has thus far not been isolated [2]. This factor increases permeability of the glomerular filtration barrier. Interestingly, Carraro et al. reported increased permeability activity in sera from five patients with *NPHS2* mutations [45]. In one patient with post RTx recurrence, the serum activity clearly correlated with proteinuria, and after commencement of plasma exchange, this activity disappeared in parallel with normalization of proteinuria. Overall, these experiments support an interaction between genetics and circulating factors in generating NS. Although the molecular basis of this remains unknown, therapy with PE and CP seems reasonable also in FSGS patients with podocin mutations and recurrence of NS after RTx.

Living-related RTx from donors bearing heterozygous *NPHS2* mutations seem not to be associated with an increased risk of proteinuria after RTx [46]. The few patients with recurrence have undergone either cadaveric or living-related RTx. The recommendation is, however, that genetic analysis of the potential donor be performed. Adult-onset SRNS has been described in patients heterozygous for a pathogenic *NPHS2* mutation together with a pR229Q variant. As this variant appears in 3.6 % of the population, the risk exists that a parent of a patient with an *NPHS2* mutation might also bear the pR229Q variant, which might later lead to proteinuria.

## Key summary points

- Severe proteinuria and NS are very rare in primary CNS patients after RTx

- The most probable etiology is NPHS1 with a gene mutation leading to absence of nephrin, as occurs in Fin-major homozygotes

- In the majority of these patients, anti-nephrin antibodies are detectable after RTx

- Sometimes NS can develop after RTx in patients with *NPHS2* mutations, but the mechanisms are still elusive

- The best treatment options known today are MP, cyclophosphamide, and plasma exchange alone or combined with Rituximab

## Conclusions

Recurrence of nephrosis in CNS after renal transplantation is extremely rare except in Finmajor/Fin-major mutations, where it appears in 30 % of patients. It has been documented in NPHS2 but not in other podocyte gene mutations. In NPHS1, anti-nephrin antibodies seem to play a role, and in NPHS2, circulating factors may be involved. In both MP, CP and PE seem to work and in NPHS1 with several episodes of re-nephrosis, Rituximab seems to lead to permanent remission. Some patients who have their re-nephrosis triggered by a severe infection or rejection may be more therapy resistant.

## Multiple-choice questions (answers are provided following the reference list)

1. The outcome of renal transplantation in infants with a genetic form of nephrotic syndrome is usually:
  - worse than in older children
  - comparable to that in older children
  - better than in older children
2. The risk for recurrence of nephrotic syndrome after transplantation in patients with a genetic kidney disease is:
  - very high
  - moderate
  - very low
3. Recurrence of nephrotic syndrome after renal transplantation is most common in patients with mutations in the:
  - nephrin gene (*NPHS1*)
  - podocin gene (*NPHS2*)
  - phospholipase c-epsilon gene (*NPHS3*)
4. Because of recurrence risk, a child with nephrin gene mutations should receive the graft from:
  - one of the parents
  - an unrelated, deceased donor
  - either a living-related or deceased donor
5. Patients with a post-transplant recurrence of nephrotic syndrome have been successfully treated with a combination of:
  - increased prednisone and anti-thymocyte globulin
  - plasma exchange and cyclophosphamide
  - increased cyclosporine and mycophenolate
